# Fertility hormones and vitamin E in active and passive adult male smokers in Calabar, Nigeria

**DOI:** 10.1371/journal.pone.0206504

**Published:** 2018-11-06

**Authors:** Iya Eze Bassey, Rebecca Mtaku Gali, Alphonsus Ekpe Udoh

**Affiliations:** 1 Department of Medical Laboratory Science, Faculty of Allied Medical Sciences, College of Medical Sciences, University of Calabar, Calabar, Cross River State, Nigeria; 2 Department of Medical Laboratory Science, College of Medical sciences, University of Maiduguri, Maiduguri, Borno State, Nigeria; Nanjing Medical University, CHINA

## Abstract

Smoking is an extremely lethal act and is associated with many illnesses. Lately, major concerns that passive smokers face the same health risks as (if not higher than) active smokers have been raised. Some studies have shown that active smoking is associated with low serum levels of vitamins and testosterone. Are these facts also valid in passive smokers? This study investigated the levels of cotinine, testosterone, follicle stimulating (FSH), Luteinizing Hormone (LH), prolactin and vitamin E in male active smokers and compare these with male passive smokers. Serum levels of cotinine, testosterone, FSH, LH, prolactin and vitamin E were determined in 60 cigarette smokers, 60 passive smokers and 60 non-smokers recruited from Calabar metropolis. The hormones were assayed using ELISA and Vitamin E using high performance liquid chromatography. Socio-demographic and anthropometric indices were obtained and data analyzed using PAWstatistic 18. Cotinine levels were significantly (p<0.05) higher in active smokers than in passive smokers and controls. Vitamin E and testosterone were significantly lower in both active (p<0.05) and passive smokers (p<0.05) when compared to non-smokers. The FSH of the active smokers was significantly higher (p = 0.034) than that of the controls while the passive smokers had the highest LH values (p = 0.0001). However, there were no significant variations in the prolactin levels among the three groups. Both passive and active smoking depletes serum vitamins E and lowers testosterone levels. Lower serum vitamin E is pointer to increased oxidative stress which in conjunction with lower testosterone levels may lead to increased incidence of infertility in both active and passive male smokers.

## Introduction

The effect of smoking tobacco in human health are serious and in many cases, deadly [1]. This practice kills about half of its users i.e. nearly 6 million people each year [2]. More than 5 million of those deaths are the results of direct tobacco use while more than 600,000 are the result of non-smokers being exposed to second-hand smoke [2]. Recently, major concerns that passive smokers face the same or even greater health risks than active smokers have been raised [3]. It is often thought that medical consequences of smoking results from direct use of tobacco products but it has been shown that passive or secondary smoke also increase the risk of many diseases [4] including ischaemic heart disease, lower respiratory infections, asthma and lung cancer. The levels of cotinine (a key metabolite of nicotine) in the blood is proportionate to the amount of exposure to tobacco smoke and therefore can be utilized as a marker for both active smoking and as an index to environmental tobacco smoke exposure [5].

Active cigarette smoking, in males, has been associated with diminished libido, impotence, erectile dysfunction and premature ejaculation, [6] and a reduction in semen quality resulting in relative infertility [3, 7]. However, there seems to be no general consensus on the effects of smoking on male reproductive hormones such as testosterone, follicle stimulating hormone (FSH), luteinizing hormone (LH), and prolactin. Some studies have reported decreased testosterone and increased FSH and LH levels in active smokers and no difference in prolactin [8,9] and in animal models [10,11]. However, some other authors have reported increased serum testosterone, LH and prolactin levels [12], while others reported that smokers and nonsmokers showed no significant differences in their mean values of reproductive hormones [13]. Pasqualotto et al. [14] did not report any significant differences in the total testosterone, LH and FSH of smokers and nonsmokers. Mitra et al. [15] and Bakheet and Amarshad [16] observed higher serum levels of FSH and LH in smokers.

Increased oxidative stress and hypoxia induced by cigarette smoking play a fundamental role in the aetiology of male infertility by negatively affecting sperm quality and function [17]. Cigarette smoke is a major cause of oxidative stress which depletes antioxidant enzymes and non-enzymatic antioxidants [18].

Antioxidant depletion has been used as a surrogate measure of oxidative stress in smokers in other studies [19]. Its use is based on the assumption that there is both consumption and depletion of endogenous antioxidants under conditions of oxidative stress and this would therefore be an indirect reflection of oxidative stress resulting from smoking. Vitamin E is known as important antioxidant to protect the reproductive system as it is is one of the best antioxidants for the removal of oxidative stress in male reproductive system [20]

There is presently a gap in knowledge as regards the effects of cigarette smoke on fertility hormones in adult male passive smokers. Most of the literature on effects of passive smoking on fertility hormones has been done on women of reproductive age [21–23]. This research work therefore was designed to investigate the relationship between tobacco smoking and serum concentrations of male reproductive hormones—luteinizing hormone (LH), follicle stimulating hormone (FSH), testosterone and prolactin and Vitamin E in both active and passive male smokers.

## Methodology

### Study design and subject selection

This was a case-control study. One hundred and eighty (180) apparently healthy male subjects aged between 18 and 45 years were consecutively recruited from different parts of Calabar metropolis for this study. They were made up of 60 active cigarette smokers, 60 passive smokers and 60 non-smokers. The active and passive smokers were mainly recruited by the research team from motor parks, cigarette smoking dens, and relaxation bars with no smoking restrictions. The controls were recruited from the University hostels, offices and through home visits. The study was carried out from 22 September 2015 to 1 September 2016. They were all Nigerians living in the Calabar metropolis of Cross River state. This study was carried out in accordance with the World Medical Association’s Declaration of Helsinki [24]. Ethical clearance was obtained from the Cross River State Ministry of Health (Ref No.: RP/REC/2015/311). The study participants were informed of the nature of the research and written informed consent obtained as approved by the ethics committee before they were enrolled for the study. The active smokers were regular smokers i.e. smokers who smoked ≥ 5 cigarette sticks per day, the passive smokers were people regularly exposed to second hand smoke at home, work or in societal gatherings while the controls had never smoked cigarettes in their life neither had they been exposed to cigarette smoke at home, work or in societal gathering. A standard questionnaire was administered to them to obtain demographic information. Each participant’s weight and height were measured. A weighing balance was utilized to measure weight in kilograms and a stadiometer was used to measure height in meters. Body mass index (BMI) was calculated by as the ratio of the weight to the square of height (kg/m^2^).

### Inclusion criteria

All the subjects were male. They had to be 18–45 years of age, Nigerians, resident in Calabar metropolis and not hospitalized as at the time of survey. The active smokers were regular smokers i.e. smokers who smoked between ≥ 5 cigarette sticks per day. The passive smokers were people regularly exposed to second hand smoke at home, work or in societal gathering. The controls had never smoked cigarettes in their life neither had they been exposed to cigarette smoke at home, work or in societal gathering.

### Exclusion criteria

All female smokers were excluded. Using the questionnaire, smokers who had been diagnosed of any smoking-related disease (such as coronary heart disease, lung cancer, etc), terminal disease or on drugs such as opium or cannabis, were excluded from the study.

### Collection of sample

Five millilitres of venous blood was aseptically collected, allowed to clot at room temperature and centrifuged at 3000rpm. Serum obtained was stored frozen until assayed.

### Estimation of male fertility hormones and cotinine

Testosterone, Luteinizing hormone, follicle stimulating hormone and prolactin levels were determined using Enzyme Linked Immunosorbent Assay kits obtained from DRG International Incorporated, East Mountain Side, USA. The tests were carried out according to the manufacturer’s instruction. Reference range for testosterone was 3 -10ng/ml and testosterone levels <3ng/ml were considered lower than normal. Cotinine was estimated using a solid-phase competitive Enzyme Linked Immunosorbent assay kit obtained from BQ kits Diagnostics, San Diego. They were estimated in the Chemical Pathology Laboratory of the University of Calabar Teaching Hospital, Calabar, Nigeria.

### Estimation of serum vitamin E

Serum Vitamin E was estimated by Waters 616/626 HPLC (Waters Ltd, United Kingdom) at the International institute of tropical Agriculture (IITA), Ibadan, Nigeria.

### Statistical analysis

Data was analyzed using the PAWstatistic 18, a statistical package from SPSS Inc, California, USA. Results were expressed as Mean ± SD. Comparisons of groups were made using analysis of variance and post hoc analysis using Least significant difference. Pearson’s correlation and regression analysis were also done. The level of significance was set at 95% confidence interval, where p-value less than 0.05 (p<0.05) was considered as statistically significant. Graphs were created with Microsoft excel 2007 version.

## Results

The socio-demographic characteristics of the study participants is shown in Table 1. A total of 180 men participated in the study comprised of 60 active and 60 passive smokers as well as 60 controls. Their mean ages were 26.2 ± 4.92 years, 26.1 ± 4.43 years and 26.4 ± 4.25 years active smokers, passive smokers and controls respesctively. Participants in this study were of the Efik, Ibibio, Ekoi, Ibo, Yoruba and Hausa tribes. Their occupations included manual and semi-skilled labour, Civil service, Military service, some were students and the rest unemployed. Majority of the participants were single. The active smokers had a mean smoking pack years of 4.08 ± 3.95 (Range 0.25–20). Sixty percent of the passive smokers were exposed daily; 36.7% were exposed 2–6 times a week and 13.3% at least once a week to second hand smoke. About 3% of the active smokers had testosterone levels <3ng/ml but none of the passive smokers or controls had testosterone levels <3ng/ml (Table 1).

**Table 1:** Sociodemographic characteristics of the study participants.

Characteristics		Active smokers n = 60	Passive smokers n = 60	Control n = 60
Age (years)	Mean	26.2	26.1	26.4
	± sd	4.92	4.43	4.25
Ethnicity n (%)	Efik	19 (31.7)	24 (40.0)	20(33.3)
	Ibibio	23 (38.3)	20 (33.3)	19(31.7)
	Ekoi	12 (20.0)	7 (11.7)	11(18.3)
	Ibo	5 (8.3)	4(6.7)	7 (11.7)
	Yoruba	1 (17.0)	5 (8.3)	2 (3.3)
	Hausa	0 (0)	0 (0)	1 (1.7)
Marital Status n (%)	Single	53 (88.3)	56 (93.3)	57 (95.0)
	Married	6 (10.0)	4 (6.7)	2 (3.3)
	Widower	1(1.7)	0 (0)	1 (1.7)
Occupation n (%)	Manual and semi-skilled labourers	18 (30.0)	21 (35.0)	49 (81.7)
	Students	17 (28.3)	24 (40.0)	11 (18.3)
	Unemployed	17 (28.3)	11 (18.3)	0 (0)
	Civil servant	6 (10.0)	4 (6.7)	0 (0)
	Military	2 (3.3)	0 (0)	0 (0)
Packets smoked per year	Mean	4.08	-	-
	± sd	3.95	-	-
Frequency of exposure of passive smokers	Daily	-	60.0%	-
	2–6 times a week	-	36.7%	-
	At least once a week	-	13.3%	-
Proportion of testosterone <3ng/ml	Frequency	2	-	0
	%	3.3	0.0	0.0

A comparison of age, body mass index, cotinine, male fertility hormones and Vitamin E in active and passive smokers and non-smokers showed a significant variation in the mean levels of cotinine, testosterone luteinizing hormone and vitamin E among the groups. There was however no significant variation in age, BMI, follicle stimulating hormone and prolactin among the groups (Table 2). Cotinine levels were significantly (p = 0.0001) higher in active smokers (41.0 ± 50.28 ng/ml) than in passive smokers (2.5 ±1.92 ng/ml) and controls (0.86 ±1.11ng/ml). Testosterone and Vitamin E were significantly lower in active (p = 0.003 and p = 0.0001) and passive smokers (p = 0.0001 and p = 0.0001) when compared to non-smokers. The active smokers had the lowest mean values of vitamin E (30.9 ± 13.17mg/ml). The FSH of the active smokers (9.4 ± 0.52mIU/ml) were significantly higher (p = 0.034) than those of the controls (6.4 ± 5.94mIU/ml) while the passive smokers had the highest (p = 0.0001) LH values (26.2 ±7.86. ng/ml). (Table 3)

**Table 2:** Comparison of age, body mass index, cotinine, male fertility hormones and vitamin E in active and passive male smokers and non-smokers.

Parameter	Active smokers	Passive smokers	Controls	Calc F	Crit F	p-value
**Age (yrs)**	26.2 ± 4.92	26.1 ± 4.43	26.4 ± 4.25	0.035	3.047	0.966
Body mass index (kg/m^2^)	24.6 ± 3.63	24.1± 1.79	24.3 ±4.06	0.290	3.047	0.748
Cotinine (ng/ml)	41.0 ± 50.28	2.5 ±1.92	0.86±1.11	36.684	3.047	0.0001
Testosterone (ng/ml)	8.4 ± 3.79	7.8 ± 2.87	10.3 ± 6.98	8.762	3.047	0.0001
Follicle stimulating hormone (mIU/ml)	9.4 ± 0.52	7.2 ± 3.47	6.4 ± 5.94	2.468	3.047	0.088
Luteinizing hormone (ng/ml)	19.2 ± 10.03	26.2 ±7.86	17.7 ± 9.72	14.686	3.047	0.0001
Prolactin (ng/ml)	9.1 ± 4.96	8.35 ± 2.27	10.3 ± 6.97	2.202	3.047	0.114
Vitamin E (mg/ml)	30.9 ± 13.17	37.1±9.20	55.4±8.70	87.687	3.047	0.0001

**Table 3:** A post hoc analysis of cotinine, male fertility hormones and vitamin E in active and passive male smokers and non-smokers.

Parameter	Groups		Mean Difference	Std Error	p-value
	Active smokers n = 60	Controls n = 60			
Cotinine (ng/ml)	41.0 ± 50.28	0.86±1.11	40.141	5.304	0.0001*
Testosterone (ng/ml)	8.4 ± 3.79	10.3 ± 6.98	-1.995	0.662	0.003*
Follicle stimulating hormone (mIU/ml)	9.4 ± 0.52	6.4 ± 5.94	2.978	1.391	0.034*
LH(ng/ml)	19.2 ± 10.03	17.7 ± 9.72	1.502	1.689	0.375
Vitamin E (mg/ml)	30.9 ± 13.17	55.4±8.70	-24.501	1.926	0.0001*
	**Passive smokers** **n = 60**	**Controls** **n = 60**			
Cotinine (ng/ml)	2.5 ±1.92	0.86±1.11	1.630	5.304	0.759
Testosterone (ng/ml)	7.8 ± 2.87	10.3 ± 6.98	-2.665	0.662	0.0001*
Follicle stimulating hormone (mIU/ml)	7.2 ± 3.47	6.4 ± 5.94	0.778	1.391	0.576
LH(ng/ml)	26.2 ±7.86	17.7 ± 9.72	8.572	1.689	0.0001*
Vitamin E (mg/ml)	37.1±9.20	55.4±8.70	-18.381	1.926	0.0001*
	**Active smokers** **n = 60**	**Passive smokers** **n = 60**			
Cotinine (ng/ml)	41.0 ± 50.28	2.5 ±1.92	38.512	5.304	0.0001*
Testosterone (ng/ml)	8.4 ± 3.79	7.8 ± 2.87	0.670	0.662	0.313
Follicle stimulating hormone (mIU/ml)	9.4 ± 0.52	7.2 ± 3.47	2.200	1.391	0.115
LH(ng/ml)	19.2 ± 10.03	26.2 ±7.86	-7.070	1.689	0.0001*
Vitamin E (mg/ml)	30.9 ± 13.17	37.1±9.20	-6.120	1.926	0.002

**Fig 1:**
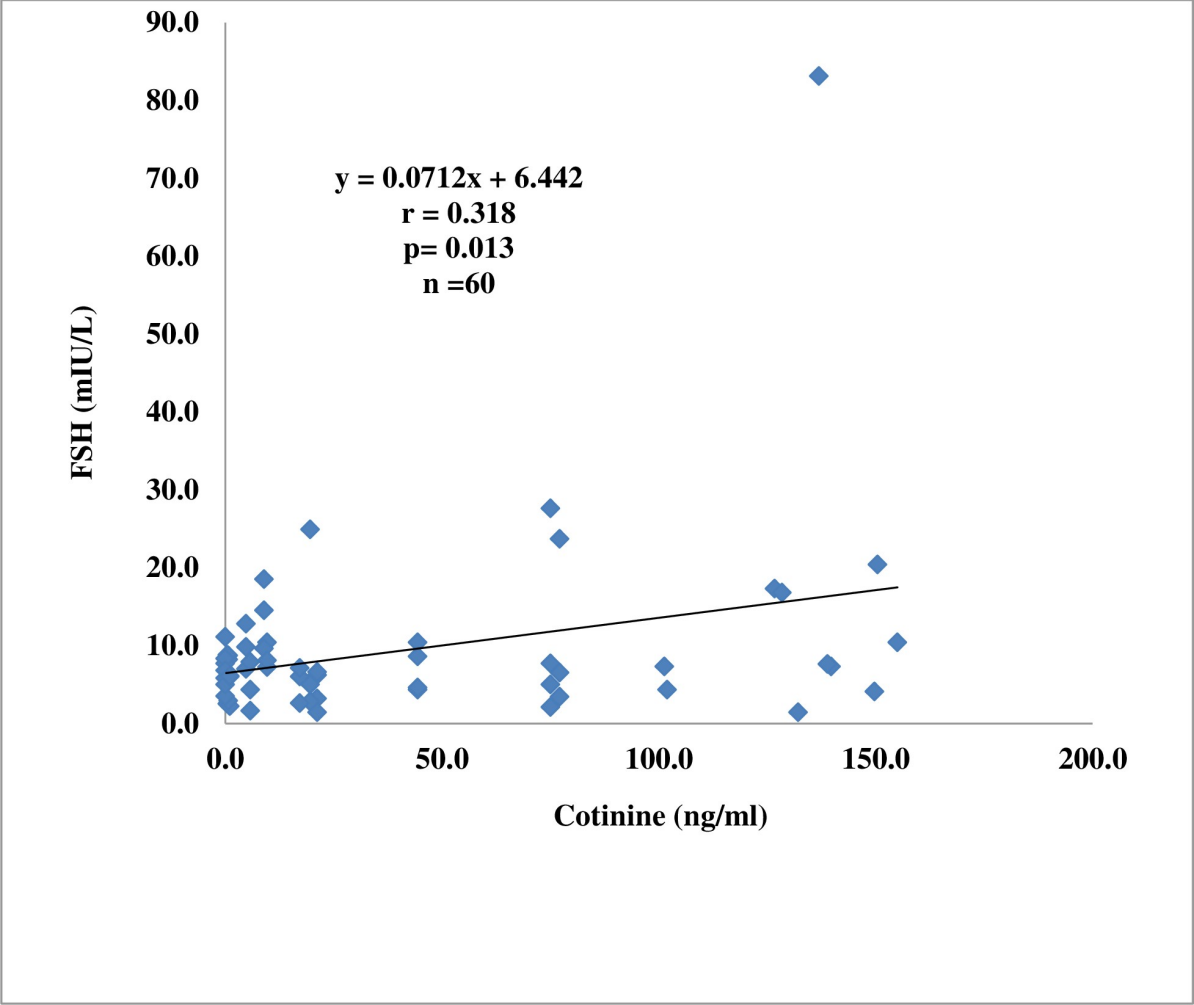
Correlation plot of follicle stimulating hormone against cotinine in active smokers.

**Fig 2:**
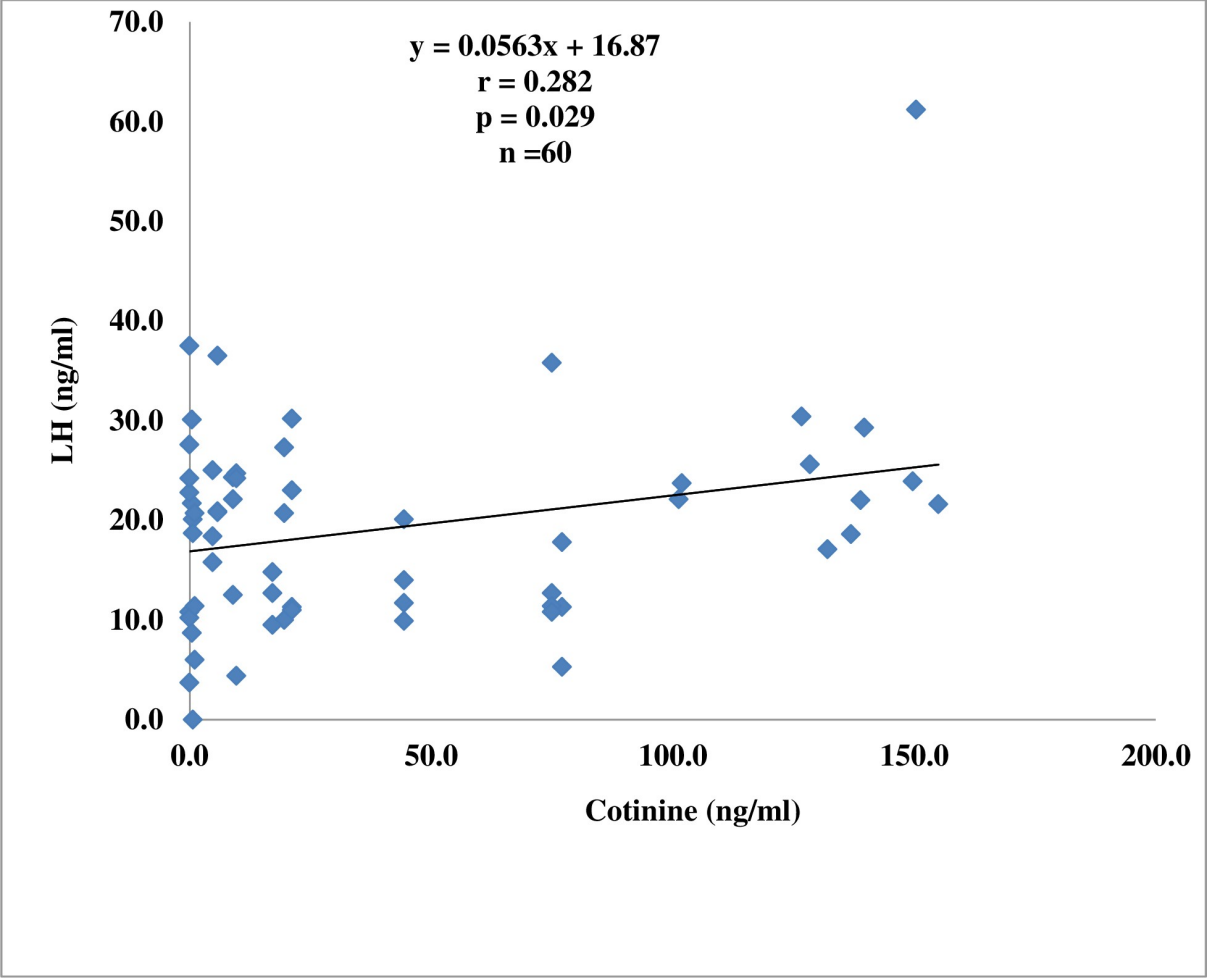
Correlation plot of luteinizing hormone against cotinine in active smokers.

There was a positive and significant correlation between FSH and cotinine (r = 0.318; p = 0.013) and between LH and cotinine (r = 0.282; p = 0.029) among the active smokers (Figs. 1 and 2).

## Discussion

One of the important indices of exposure to environmental tobacco smoke that can also be utilized as a marker for active smoking is cotinine [5]. In this study, the cotinine levels of the active smokers showed that they had the highest levels of exposure to tobacco smoke compared to passive smokers and controls. This is because active smokers are exposed to deeply inhaled mainstream smoke as well as sidestream smoke [25], while passive smokers are exposed to only the less concentrated and dilute environmental tobacco smoke [26]. In spite of this, the harmful effects of smoking on reproductive hormones were observed in both groups of smokers as shown by lower testosterone and Vitamin E levels and higher FSH and LH levels compared to the controls.

Smoking has been known to increase hepatic metabolism of testosterone which may consequently lead to a reduced testosterone level in serum [3] which may account for the lower testosterone levels observed in active and passive smokers in this study. These lower levels of testosterone in both active and passive smokers may translate to a relative decrease in fertility because testosterone is a very vital male reproductive hormone. Though both groups had lower testosterone, it was only among the active smokers that we observed that 3% of them had testosterone levels below 3 ng/ml which is suggestive of hypogonadism in these men and the fact that the effects of smoking in the active smokers may be more acute than in passive smokers. This is probably due to the higher exposure to nicotine in active smokers. This agrees with findings by [8, 27].

The higher levels of LH in the passive smokers and FSH in the active smokers observed in this study are indicators of testicular dysfunction or gonadal failure [28]. Elevated levels of these two hormones in males usually reflect lack of male sex steroid hormone negative feedback [29]. Saadat [30] also reported similar findings in male smokers. The positive correlation between FSH and cotinine, and LH and cotinine in the active smokers observed in this study indicates a dose-response relationship between exposure to cigarette smoke and testicular dysfunction.

Several studies have documented the damaging effects of smoking on the antioxidant defense system due to increased oxidative stress [7, 17]. Cigarette smoke contains high concentrations of free radical generating molecules such as nitric oxide (NO) and quinones which consequently result in the production of high levels of toxic metabolites such as reactive nitrogen and oxygen species [18]. The consequence of all these is oxidative stress and the resultant depletion of Vitamin E. Vitamins E is a lipid soluble vitamin with antioxidant properties that prevents lipid peroxidation by reacting with free radicals such as the peroxyl radicals as well as with singlet molecular oxygen [31]. Some studies have reported low vitamin E in infertile males [32, 33] and suggested that oxidative stress/lipid peroxidation is associated with infertility [34].

In our study there were no differences in the prolactin levels of either the active or passive smokers compared to the non smokers. However, some studies have associated increased levels of prolactin with cigarette smoking in male smokers [12, 27] whilst others have reported lower levels of prolactin in smokers [35]. This was not the case in our study, though prolactin was lower in both groups of smokers compared to the controls, these were not significant.

A limitation of this study was that additional antioxidants and antioxidant enzymes were not assayed to ascertain the total extent of damage to the antioxidant system especially in passive smokers.

This study has shown that despite differences in the levels of exposure, in both passive and active smokers, there is a disruption in the hormonal balance of the sex hormones (especially testosterone) and depletion of Vitamin E. This may lead to a decline in semen quality, which is very vital in determining the chances of conception as it is usually regarded as a proxy measure of fertility in males. This is in agreement with the WHO stand that “there would be no safe level of human exposure to tobacco second hand smoke” [1].

## Conclusion

Both passive and active smoking depletes serum vitamins E and lowers testosterone levels. Lower vitamin E is pointer to increased oxidative stress which in conjunction with lower testosterone levels may lead to increased incidence of infertility in both active and passive male smokers.

## Supporting information

S1 Data (SAV)
